# Supplementary material for: Clinical Implications of Serum Hepatitis B Virus Pregenomic RNA Kinetics in Chronic Hepatitis B Patients Receiving Antiviral Treatment and Those Achieving HBsAg Loss
Source: Microorganisms. 2021 May 26;9(6):1146. doi: 10.3390/microorganisms9061146 (PMC8229518; doi:10.3390/microorganisms9061146)
Supplement: Supplementary file 1 [file microorganisms-09-01146-s001.zip › Supplementary Figures.pdf]

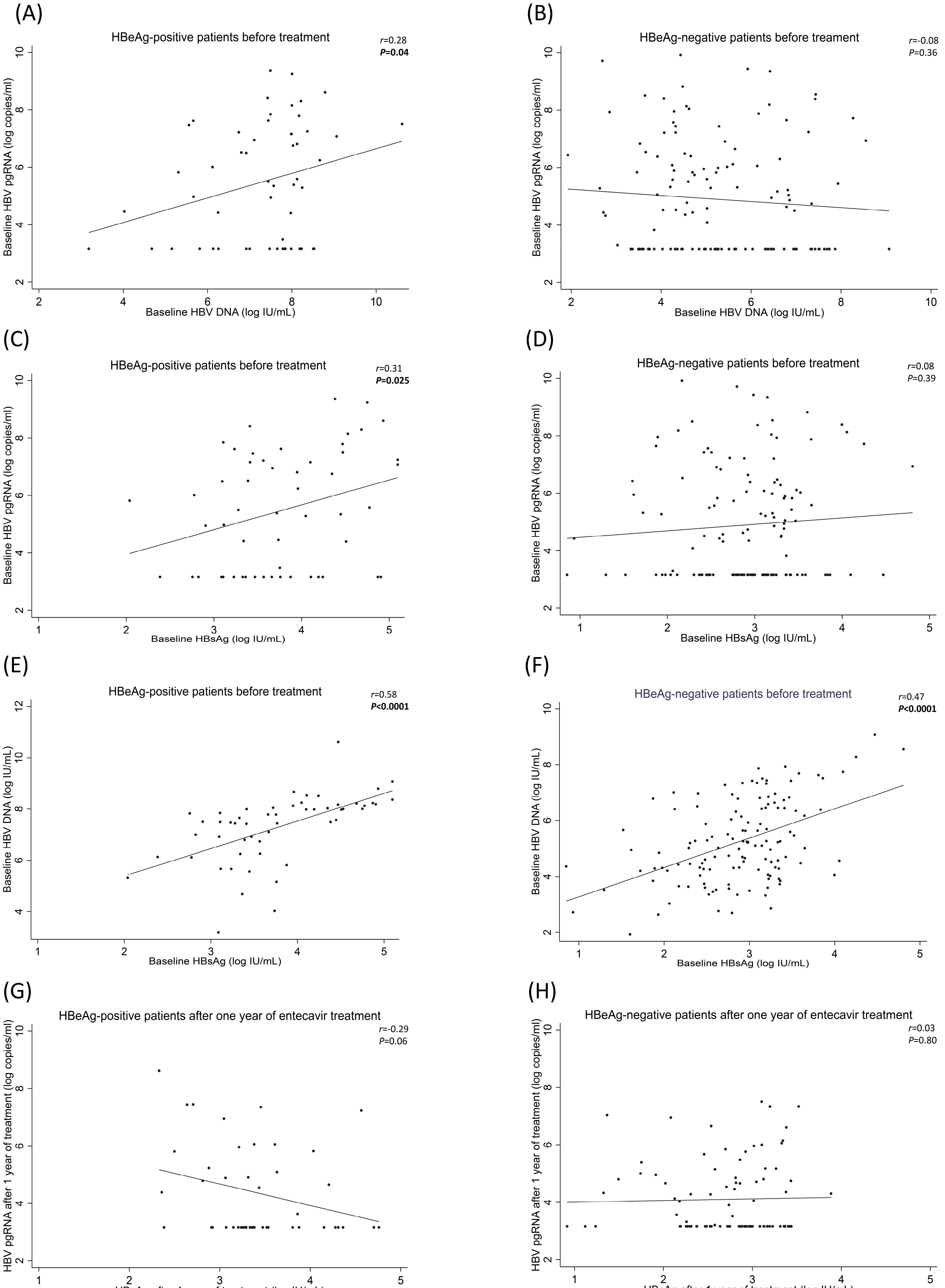

Figure S1. Correlation between serum HBV pgRNA, HBV DNA, and HBsAg. Correlation between HBV pgRNA and HBV DNA before treatment in HBeAg-positive (A) and HBeAg-negative patients (B). Correlation between HBV pgRNA and HBsAg before treatment in HBeAg-positive (C) and HBeAg-negative patients (D). Correlation between HBV DNA and HBsAg before treatment in HBeAg-positive (E) and HBeAg-negative patients (F). Correlation between serum HBV pgRNA and HBsAg after one year of entecavir treatment in HBeAg-positive (G) and HBeAg-negative patients (H). Significant *P* values are presented in bold.

(A)

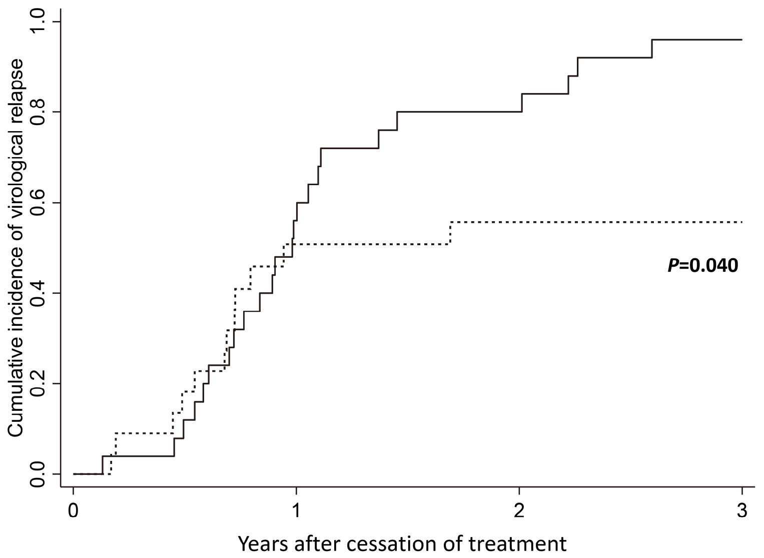

Number at risk				
HBV pgRNA ≥1466	25	11	5	1
HBV pgRNA <1466	22	10	9	7

——— HBV pgRNA ≥1466 copies/mL at the end of treatment

········· HBV pgRNA <1466 copies/mL at the end of treatment

(B)

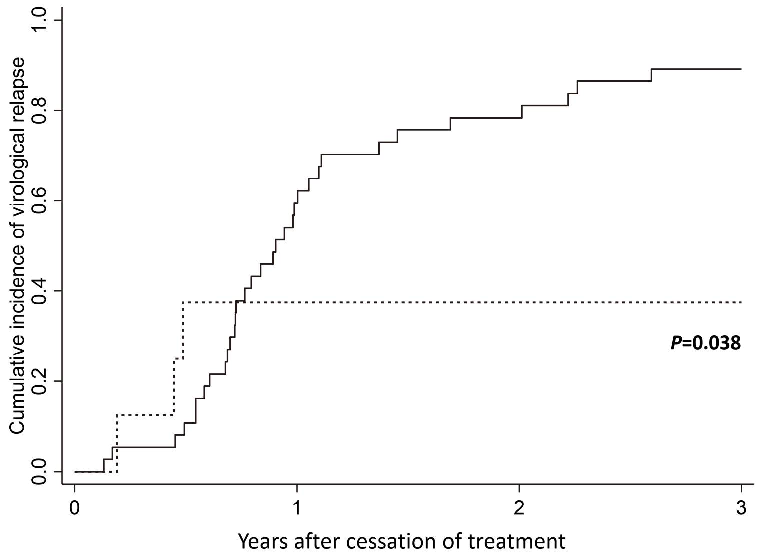

Number at risk				
HBV pgRNA ≥1466	37	15	8	4
HBV pgRNA <1466	8	5	5	3

——— HBsAg ≥2 log IU/mL or HBV pgRNA ≥1466 copies/mL at the end of treatment

········· HBsAg <2 log IU/mL and HBV pgRNA <1466 copies/mL at the end of treatment

Figure S2. Cumulative incidence of virological relapse after cessation of entecavir therapy, categorized by “HBV pgRNA ≥ 1466 copies/mL” (A) and “HBsAg ≥ 2 log IU/mL or HBV pgRNA ≥ 1466 copies/mL” (B) at the end of treatment.

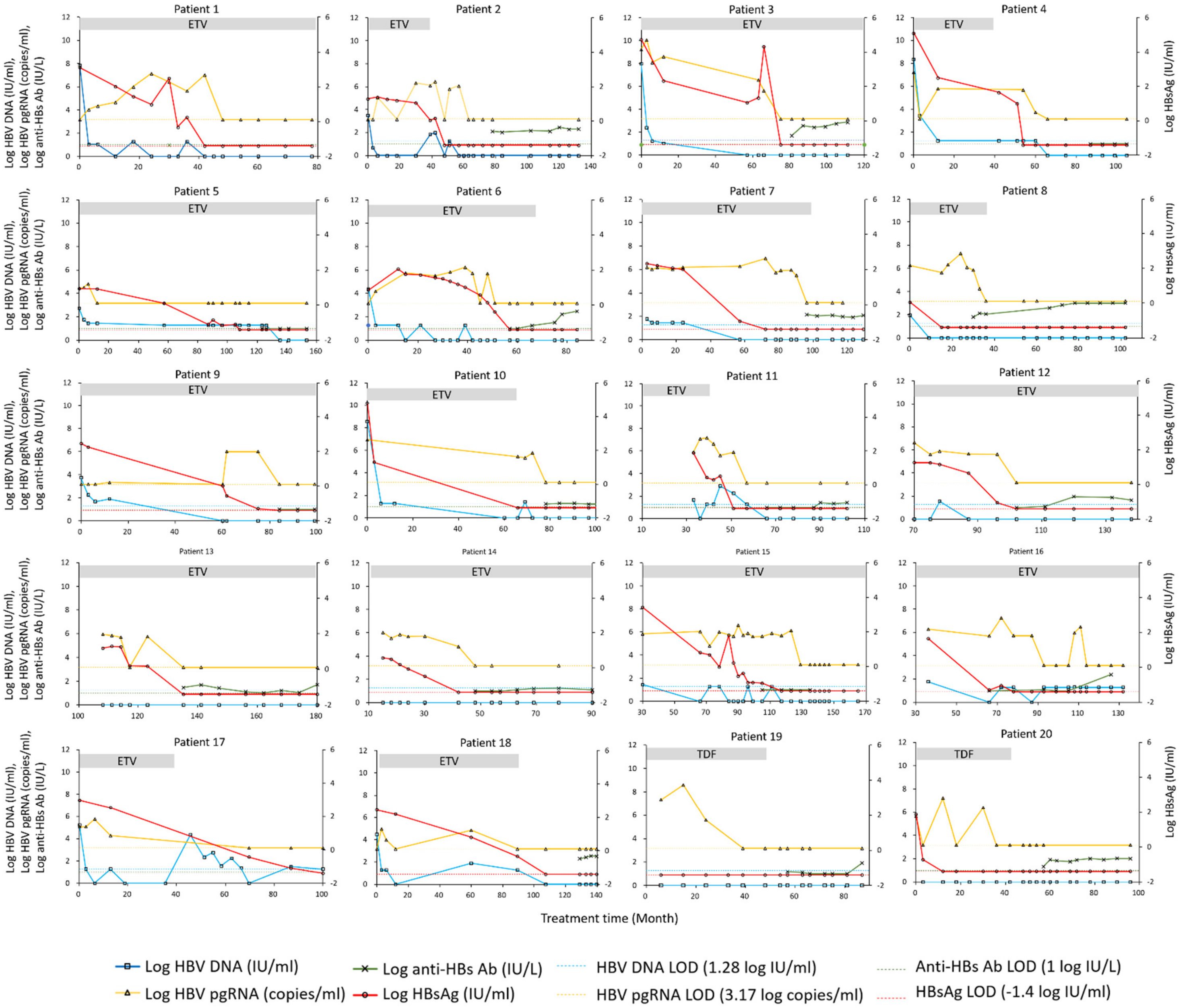

Figure S3. HBV pgRNA kinetics in 20 chronic hepatitis B patients who achieved HBsAg loss after nucleos(t)ide analogue treatment. The HBsAg samples with values below LOD (0.05 IU/mL) or HBsAg not detected were recorded as 0.04 IU/mL (−1.40 log IU/mL). The HBV DNA samples with values below LOD and the serum samples in which HBV DNA was not detected were recorded as LOD − 1 IU/mL and 1 IU/mL, respectively. The HBV pgRNA samples with values below LOD (1466 copies/mL) were recorded as 1465 copies/mL (3.17 log copies/mL). HBV pgRNA were detected, although some were below LOD, in all tested serum samples of the 20 patients before and after HBsAg loss.
