# Supplementary material for: Clinical Implications of Serum Hepatitis B Virus Pregenomic RNA Kinetics in Chronic Hepatitis B Patients Receiving Antiviral Treatment and Those Achieving HBsAg Loss
Source: Microorganisms. 2021 May 26;9(6):1146. doi: 10.3390/microorganisms9061146 (PMC8229518; doi:10.3390/microorganisms9061146)
Supplement: Supplementary file 1 [file microorganisms-09-01146-s001.zip › Supplementary Tables.pdf]

Table S1. Primers for HBV pgRNA amplification and detection

Round	Orientation[a]	Primer name	Sequence (5'-3')	Position
RT	-	HBV-3.5RNA-RT	ATTCTCAGACCGTAGCACACGACACCGAGATTGAGATCTTCTGCGAC	nt 2436-2415
PCR	+	3.5pgRNA_PCR-F	CCTACTGTTCAAGCCTCCAAGC	nt 1856-1877
	-	HBV RNA_PCR-R	ATTCTCAGACCGTAGCACACGACAC	random sequence
QPCR	+	3.5RNA_QPCR-F	AYAGACCATCAAATGCCC	nt 2295-2312
	-	HBV RNA_PCR-R	ATTCTCAGACCGTAGCACACGACAC	random sequence

[a] Orientation of primer sequence (+, sense; - antisense)

Table S2. The limit of detection for serum HBV pgRNA was 1466 copies/mL, as calculated by probit analysis

**Nominal input** (HBV pgRNA copies/mL)	**No. replicates**	**No. positives**	**Positivity rate (%)**
19200	24	24	100
1920	60	58	96.67
960	80	77	96.25
660	80	17	21.25
480	80	7	8.75
240	60	4	6.67
The concentration of HBV pgRNA that can be detected with a positivity rate of greater than 95% as determined by probit analysis is 1466 copies/mL			

Table S3. Clinical characteristics of 185 chronic hepatitis B patients for studying the kinetics of serum HBV pgRNA during entecavir therapy, categorized by baseline HBV pgRNA levels

Characteristics	**Baseline HBV pgRNA ≥6 log copies/mL** (n=61)	**Baseline HBV pgRNA ≥4 and <6 log copies/mL** (n=44)	**Baseline HBV pgRNA <4 log copies/mL** (n=80)	***P***
Age (year)	48.7 ± 12.8	53.4 ± 11.3	51.5 ± 11.6	0.12
Male (%)	41/61 (67.2%)	33/44 (75%)	56/80 (70%)	0.69
HBeAg-positive patients (%)	24/61 (39.3%)	11/44 (25%)	19/80 (23.8%)	0.10
HBV genotype (B:C)[a]	29:25	20:18	39:33	0.99
Baseline ALT (× ULN)	4.45 ± 7.57	2.85 ± 3.70	3.92 ± 5.27	0.38
Liver cirrhosis (%)	16/61 (26.2%)	19/44 (43.2%)	27/80 (33.8%)	0.19
HCC (%)[b]	7/61 (11.5%)	9/44 (20.5%)	13/80 (16.3%)	0.45

Continuous variables are expressed as mean ± standard deviation, except time to virological response, time to HBeAg seroconversion, and time to virological response and HBeAg seroconversion, which are expressed as median (range). Significant p values are presented in bold.

[a] HBV genotype was not determined in 21 patients because of low baseline HBV viral loads.

[b] HCC (hepatocellular carcinoma) diagnosed before or within half a year of entecavir therapy.

Table S4. Clinical characteristics of 47 patients with chronic hepatitis B for analysis of the factors associated with virological relapse after cessation of entecavir therapy, categorized by baseline HBeAg status

**Characteristics**	**Total patients** (n=47)	**HBeAg-positive patients** (n=26)	**HBeAg-negative patients** (n=21)	***P***[a]
Age at baseline (year)[b]	42.7 ± 11.8	35.9 ± 10.2	51.1 ± 7.7	**<0.0001**
Male (%)	35/47 (74.5%)	20/26(76.9%)	15/21 (71.4%)	0.67
HBV genotype (B:C)[c]	20:24	8:16	12:8	0.08
Baseline ALT (× ULN)[b,d]	5.62 ± 7.05	4.78 ± 5.83	6.63 ± 8.33	0.39
Baseline HBsAg (log IU/mL)[b,e]	3.51 ± 0.98	4.11 ± 0.55	3.07 ± 1.01	**0.017**
Baseline HBV DNA (log IU/mL)[b,f]	6.55 ± 1.58	7.37 ± 1.12	5.60 ± 1.52	**0.0001**
Liver cirrhosis at baseline (%)[b]	4/47 (8.5%)	2/26 (7.7%)	2/21 (9.5%)	1.00
Treatment time (year)	4.32 (2.00–8.22)	4.40 (2.00–8.22)	3.48 (2.60–5.63)	0.28
Age at the end of treatment (year)	46.8 ± 11.9	40.3 ± 10.7	54.9 ± 7.6	**<0.0001**
HBeAg-positive at the end of treatment (%)[g]		4/25 (16%)		
ALT at the end of treatment (× ULN)	0.74 ± 0.85	0.55 ± 0.27	0.99 ± 1.21	0.12
HBsAg at the end of treatment (log IU/mL)[h]	2.76 ± 1.30	3.27 ± 0.71	2.19 ± 1.57	**0.007**
HBV DNA at the end of treatment (log IU/mL)	0.00 (0.00–1.84)	0.00 (0.00–1.28)	0.00 (0.00–1.84)	0.67
HBV pgRNA at the end of treatment (log copies/mL)	3.90 ± 0.97	3.98 ± 1.07	3.81 ± 0.85	0.56
Virological relapse (%)[i]	36/47 (76.6%)	20/26 (76.9%)	16/21 (76.2%)	1.00
Time to virological relapse time (year)[i]	0.78 (0.13–2.60)	0.81 (0.13–2.60)	0.78 (0.45–2.26)	0.86

Continuous variables are expressed as mean ± standard deviation, except for treatment time, HBV DNA at the end of treatment, and time to virological response, which are expressed as median (range). Significant *P* values are presented in bold.

[a] *P* values compared between HBeAg-positive patients and HBeAg-negative patients.

[b] Baseline means the time before receiving entecavir treatment.

[c] HBV genotype was not determined in two HBeAg-positive patients and one HBeAg-negative patient because of low baseline HBV viral loads or no baseline serum samples in these patients.

[d] Baseline ALT was not determined in two HBeAg-positive patients and one HBeAg-negative patient because of limited serum samples.

[e] Baseline HBsAg was not determined in 18 HBeAg-positive patients and 10 HBeAg-negative patients because of limited serum samples.

[f] Baseline HBV DNA was not determined in four HBeAg-positive patients and two HBeAg-negative patients because of limited serum samples.

[g] HBeAg at the end of treatment was not determined in one HBeAg-positive patient because of limited serum sample.

[h] HBsAg at the end of treatment was not determined in two HBeAg-positive patients because of limited serum samples.

[i] Virological relapse means serum HBV DNA > 2000 IU/mL after cessation of entecavir therapy.

Table S5. Univariate and multivariate analyses of factors associated with virological relapse after cessation of entecavir therapy

Factors	Univariate Analysis			Multivariate Analysis I			Multivariate Analysis II		
	HR	95% CI	*p*	HR	95% CI	*p*	HR	95% CI	*p*
Age ≥45 years old at the end of treatment	1.90	0.96–3.74	0.063	1.74	0.69–4.38	0.24	1.85	0.75–4.57	0.18
Sex (male *vs.* female)	0.86	0.41–1.79	0.69	1.73	0.53–0.64	0.36	1.88	0.61–5.80	0.27
HBeAg-positive at baseline	1.03	0.53–1.99	0.93	1.02	0.36–2.88	0.96	1.07	0.39–2.94	0.90
HBV genotype (C *vs.* B)	1.46	0.72–2.97	0.30	1.08	0.43–2.73	0.87	1.14	0.47–2.73	0.77
Liver cirrhosis at baseline	0.37	0.09–1.55	0.18	0.42	0.09–2.02	0.28	0.40	0.08–1.96	0.26
Treatment time (year)	1.27	0.98–1.65	0.074	1.03	0.70–1.51	0.89	0.99	0.67–1.45	0.95
HBeAg-positive at the end of treatment	2.15	0.74–6.20	0.16	2.22	0.43–11.32	0.34	2.39	0.47–12.08	0.29
ALT at the end of treatment (× ULN)	0.93	0.59–1.47	0.75	0.97	0.54–1.73	0.91	1.00	0.56–1.77	0.99
HBsAg ≥2 log IU/mL at the end of treatment	2.57	0.90–7.37	0.078	2.87	0.74–11.64	0.14			
HBV pgRNA ≥1466 copies/mL at the end of treatment	2.07	1.02–4.20	**0.044**	1.21	0.49–3.02	0.68			
HBsAg ≥2 log IU/mL or HBV pgRNA ≥1466 copies/mL at the end of treatment	3.32	1.00–10.99	**0.049**				4.26	1.08–16.89	**0.039**

Significant *P* values are presented in bold. Abbreviations: ALT, alanine aminotransferase; CI, confidence interval; HBeAg, hepatitis B e antigen; HBsAg, hepatitis B surface antigen; HBV, hepatitis B virus; HR, hazard ratio; pgRNA, pregenomic RNA; ULN, upper limit of normal.

Table S6. Clinical characteristics of 55 chronic hepatitis B patients who achieved HBsAg loss, 20 healthy subjects with negative HBsAg but positive anti-HBc, and 17 healthy controls with negative HBsAg and negative anti-HBc for assessing the expression of serum HBV pgRNA after HBsAg loss

Characteristics	CHB patients achieved HBsAg loss after nucleos(t)ide analogue treatment<br>HBsAg (-), Anti-HBc (+) (N=34)	CHB patients achieved HBsAg loss spontaneously<br>HBsAg (-), Anti-HBc (+) (N=21)	Healthy subjects with negative HBsAg but positive anti-HBc<br>HBsAg (-), Anti-HBc (+) (N=20)	Healthy controls with negative HBsAg and negative anti-HBc<br>HBsAg (-), Anti-HBc (-) (N=17)
Age (year)[a]	55.47 ± 10.67	57.60 ± 12.54	49.4 ± 9.9	36.5 ± 9.0
Male (%)	29/34 (85.3%)	12/21 (57.1%)	9/20 (45%)	7/17 (41.2%)
Liver cirrhosis (%)	5/34 (14.7%)	0/21 (0%)		
HCC (%)[b]	1/34 (2.9%)	0/21 (0%)		
HBeAg-positive before treatment (%)	7/34 (20.6%)			
Treatment history				
ETV	22			
LAM	4			
ADV	1			
TDF	1			
LAM+ADV	1			
LAM/LAM+ADV/TDF	1			

LAM/ ADV/LAM+ADV/TDF	1			
LAM/ADV/ETV	1			
LAM/ETV	1			
ADV/LAM+ADV/LAM+TDF/TDF	1			
Treatment time to HBsAg loss (year)	6.4 (1.4–14.9)			
ALT (× ULN)[c]	0.80 ± 0.73	0.69 ± 0.51	0.57 ± 0.28	0.74 ± 0.53
HBV DNA[c]				
not detected (%)	29/34 (85.3 %)	12/21 (57.1%)	20/20 (100%)	17/17 (100%)
<20 IU/mL (%)	4/34 (11.8%)	5/21 (23.8%)		
≥20 IU/mL (%)	1/34 (2.9%)<br>1.5 log IU/mL	4/21 (19.0%)<br>1.3, 1.5, 1.8, 2.1 log IU/mL		
HBV pgRNA[d]				
not detected (%)	17/34 (50%)	21 (100%)	20/20 (100%)	17/17 (100%)
<LOD (1466 copies/mL) (%)	7/34 (20.6%)			
≥LOD (1466 copies/mL) (%)	10/34 (29.4%)<br>5.80 (4.81–7.34) log copies/mL			

Continuous variables are expressed as mean ± standard deviation, except for treatment time and HBV pgRNA at or after HBsAg loss, which are expressed as median (range). Abbreviations: ADV, adefovir; ALT, alanine aminotransferase; Anti-HBc, antibody to hepatitis B core antigen; CHB, chronic hepatitis B; ETV, entecavir; HBeAg, hepatitis B e antigen; HBsAg, hepatitis B surface antigen; HBV, hepatitis B virus; HCC, hepatocellular carcinoma; LAM, lamivudine; LOD, limit of detection; pgRNA, pregenomic RNA; TDF, tenofovir disoproxil fumarate; ULN, upper limit of normal.

[a] Age at the time when the patients achieved HBsAg loss

[b] HCC was diagnosed before nucleos(t)ide analogue therapy in one patient.

[c] ALT and HBV DNA were determined at the time when the patients achieved HBsAg loss.

[d] HBV pgRNA was determined at the time when the patients achieved HBsAg loss for those with serum samples at HBsAg loss or after the time when the patients achieved HBsAg loss for those without serum samples at HBsAg loss but with serum samples after HBsAg loss.
